# The Strength and Timing of the Mitochondrial Bottleneck in Salmon Suggests a Conserved Mechanism in Vertebrates

**DOI:** 10.1371/journal.pone.0020522

**Published:** 2011-05-31

**Authors:** Jonci N. Wolff, Daniel J. White, Michael Woodhams, Helen E. White, Neil J. Gemmell

**Affiliations:** 1 School of Biological Sciences, University of Canterbury, Christchurch, Canterbury, New Zealand; 2 School of Biotechnology and Biomolecular Sciences, University of New South Wales, Sydney, New South Wales, Australia; 3 Department of Anatomy and Structural Biology, Centre for Reproduction and Genomics, University of Otago, Dunedin, Otago, New Zealand; 4 School of Information Technology, University of Sydney, Sydney, New South Wales, Australia; 5 National Genetics Reference Laboratory (Wessex), Salisbury District Hospital, Salisbury, Wiltshire, United Kingdom; Natural History Museum of Denmark, Denmark

## Abstract

In most species mitochondrial DNA (mtDNA) is inherited maternally in an apparently clonal fashion, although how this is achieved remains uncertain. Population genetic studies show not only that individuals can harbor more than one type of mtDNA (heteroplasmy) but that heteroplasmy is common and widespread across a diversity of taxa. Females harboring a mixture of mtDNAs may transmit varying proportions of each mtDNA type (haplotype) to their offspring. However, mtDNA variants are also observed to segregate rapidly between generations despite the high mtDNA copy number in the oocyte, which suggests a genetic bottleneck acts during mtDNA transmission. Understanding the size and timing of this bottleneck is important for interpreting population genetic relationships and for predicting the inheritance of mtDNA based disease, but despite its importance the underlying mechanisms remain unclear. Empirical studies, restricted to mice, have shown that the mtDNA bottleneck could act either at embryogenesis, oogenesis or both. To investigate whether the size and timing of the mitochondrial bottleneck is conserved between distant vertebrates, we measured the genetic variance in mtDNA heteroplasmy at three developmental stages (female, ova and fry) in chinook salmon and applied a new mathematical model to estimate the number of segregating units (*N_e_*) of the mitochondrial bottleneck between each stage. Using these data we estimate values for mtDNA Ne of 88.3 for oogenesis, and 80.3 for embryogenesis. Our results confirm the presence of a mitochondrial bottleneck in fish, and show that segregation of mtDNA variation is effectively complete by the end of oogenesis. Considering the extensive differences in reproductive physiology between fish and mammals, our results suggest the mechanism underlying the mtDNA bottleneck is conserved in these distant vertebrates both in terms of it magnitude and timing. This finding may lead to improvements in our understanding of mitochondrial disorders and population interpretations using mtDNA data.

## Introduction

Mitochondrial DNA (mtDNA) is the linchpin of modern population and evolutionary genetics. It is widely used to examine the evolutionary history of numerous species and has been employed to determine, for example, the origins and the global expansion of modern humans. The power of mtDNA analyses derives from the apparent simplicity of mitochondrial inheritance (maternal, clonal and without recombination), which has enabled models of population history to be much simpler than those needed for the analysis of nuclear DNA. However, a large body of evidence from population genetic studies, shows not only that individuals can harbor more than one type of mtDNA (heteroplasmy), but that it is apparently widespread in humans [1] and other eukaryotes [2].

While the extent of mtDNA heteroplasmy poses problems for data interpretation in population genetics and forensics [2]; clarifying how mtDNA heteroplasmy is maintained and inherited is particularly important for the growing list of human diseases with severities that depend upon the ratio of wild-type to aberrant mitochondria [1], [4], [5]. The number of mtDNA genomes that pass from one generation to the next is also important for assessing the rate with which mtDNA recombination may spawn new haplotypes [2], [3].

Despite almost two decades of accumulated data, the stability of mtDNA heteroplasmy across generations remains contentious. Work in cattle, humans and mice suggests that mtDNA heteroplasmy resolves rapidly to a clonal state through a genetic bottleneck at embryogenesis [6], [7], [8], [9] possibly aided by the added pressure of selection [10], [11]. However, other work suggests that mtDNA heteroplasmy is stably maintained for multiple generations [2], [12], [13], [14], [15], [16], [17], [18]. The likely driver of these differences are selection [11] and the size and thus strength of the mtDNA bottleneck, but despite its importance as a predictor of the likely pattern of mtDNA inheritance the size and timing of this event has been rarely studied.

This bottleneck has risen to recent prominence [1] due to its importance in understanding the intergenerational transmission of mitochondrial DNA mutations, which impacts upon our ability to predict the inheritance of mitochondrial disease states and make evolutionary interpretations using mitochondrial DNA data. While the size (number of segregating mtDNA molecules) of this bottleneck has been estimated for a few species [2], data to determine at what developmental stage, oogenesis or embryogenesis, such a bottleneck may occur has thus far been restricted to a few studies on mice [8], [19], [20], [21]. These studies have reported highly diverging results, leaving the issue of the developmental timing of the bottleneck equivocal and contentious in this species [1], [2], [22], [23].

**Figure 1:**
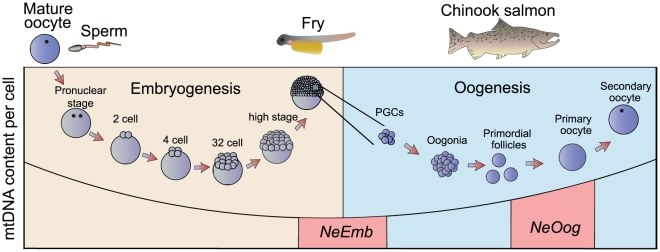
Schematic illustration of two developmental genetic bottlenecks proposed to impact on mitochondrial inheritance. A bottleneck in the female germ line has been proposed to be caused without the physical reduction of mtDNA content per cell but rather by relaxed amplification of a subset of the mtDNA population per cell (NeOog) [21]. The bottleneck during embryogenesis has been suggested to occur via random partitioning of mitochondria in the cleaving embryo resulting in a physical bottleneck at the early blastocyst stage (NeEmb) [20].

Evidence from mouse suggests that a mtDNA bottleneck acts during the early stages of embryonic development [8], [20], [24], [25]. The hypothesized role of this bottleneck is to remove mitochondrial mutations and avert ‘mutational meltdown’ in the genome of this crucial organelle [2], [24], [26], [27], [28]. However, whether this bottleneck occurs during oogenesis (*i.e.* during the development of mature oocytes from primordial germ cells, Fig. 1), embryogenesis (*i.e.* in the cleaving embryo from the zygote to the establishment of the germ layers including the primordial germ cells, Fig. 1), or both remains uncertain [8], [19], [20], [21], [25], [29].

Several studies have aimed to examine the intergenerational transmission of heteroplasmy levels between heteroplasmic mother/offspring pairs, to estimate the effective number (*N_e_*) of mtDNA transmitted from mother to offspring [7], [12], [19], [20], [21], [25], [30], [31], [32], [33], [34]. These studies have either used a direct approach by measuring the mtDNA content of single cells [19], [20], [21], [25], but more frequently have monitored differences in haplotype frequencies between mother and offspring [7], [12], [20], [30], [31], [32], [34], [35], [36]. However, to date, only two studies (both in mouse) have aimed to monitor changes in heteroplasmy levels during different developmental stages of embryogenesis and oogenesis [8], [21], and both studies excluded unequal partitioning of mtDNA during embryogenesis from contributing significantly to the observed bottleneck effects. These results are, however, contradicted by another recent mouse study that employed a direct approach [20]. Thus, the timing and strength of the bottleneck remains uncertain [discussed in 2], [20], [21], [22], [23], [24], [37]. Further, there is currently no empirical data on when this bottleneck might occur for anything other than mouse and further work on evolutionary divergent taxa is needed.

A detailed knowledge of the mitochondrial bottleneck, particularly its magnitude, timing and putative mode of action, and the evolutionary conservation of the above, has important implications for the understanding of the transmission and intergenerational stability of mitochondrial heteroplasmy [2], which impacts in areas spanning the aetiology of mitochondrial disorders [26], including some cancers [38], through to the use of mtDNA data for population and evolutionary interpretations [2].

In this study, we follow the intergenerational transmission of mtDNA in naturally occurring heteroplasmic individuals of New Zealand chinook salmon (*Oncorhynchus tshawytscha*). Using this system we have measured (i) the change in heteroplasmy levels from mother to oocytes, in order to estimate the effective number of segregating units at oogenesis, (*N_e_Oog*, Fig. 1), and (ii) the change in heteroplasmy levels from mother to offspring, in order to estimate the effective number of segregating units at embryogenesis, (*N_e_Emb*, Fig. 1). Collectively, these approaches enable us to examine the magnitude and developmental stages during which an mtDNA bottleneck is felt in a non-mammalian system, and may help us determine to what extent the mechanism underlying the bottleneck might be conserved across taxa.

## Materials and Methods

### Markers

Two heteroplasmic sites were investigated in this work, previously discovered in a hatchery population of New Zealand chinook salmon at the Silverstream hatchery, Canterbury, NZ. Both sites confer synonymous changes and are located in the mitochondrial gene *mt-nd1*, at nucleotide positions 4149 and 4316 (NCBI:NC_002980) [39]. An A/G polymorphism (A = wildtype) was found at position 4149 (A4149G), and a C/T polymorphism (T = wildtype) at position 4316 (T4316C). The A/G polymorphism at this position lies at the edge of a poly-G tract, which is likely to be unstable promoting the conversion of the A to a G and thus a high mutation rate. Heteroplasmic sites were detected by Sanger sequencing, and authenticated by cloning experiments to resolve both haplotypes.

### Samples and DNA extractions

Heteroplasmy levels in the somatic tissue (fin clips) of five female fish, and multiple oocytes (N = 13 to 28) and offspring (1–2 mm of tail tissue, N = 20) of each female were examined. Two females were heteroplasmic for nucleotide position 4316 (families 272, 357), and three were heteroplasmic for position 4149 (individual 214, 256, 263). Heteroplasmic offspring were generated by crossing homoplasmic males and heteroplasmic females using the dry method [40]. Fertilized eggs were incubated and embryos reared until hatching, following standard husbandry procedures [40], [41]. Both embryos and eggs were harvested and stored in 80% EtOH at −20°C until analysis. DNA was extracted following standard protocols using 350 µl lysis buffer (5% Chelex-100, 100 mM NaCl, 50 mM Tris [pH 8.0], 1% SDS, 10 mM EDTA, 100 µg/ml RNase A, 100 µg/ml Proteinase K) for somatic tissues and 1500 µl roe-specific lysis buffer for oocytes [42].

### Sampling strategy

We harvested oocytes from five females and measured heteroplasmy levels within multiple oocytes from each female. The variance in heteroplasmy levels among oocytes in comparison to the heteroplasmy level in the somatic tissues of the female (the oocyte donor) allows for the modelling of the segregation of mtDNA variants (*N_e_Oog*) from the beginning of oogenesis (assumed to be equivalent to the heteroplasmy levels found in the soma and gonadal structures) to the end of oogenesis (heteroplasmy level of oocytes). A subset of oocytes from the same donor was reared until hatching and heteroplasmy levels determined in these offspring. The variance in heteroplasmy levels among these offspring in comparison to the heteroplasmy level in the somatic tissues of the female allow for the modelling of mtDNA segregation between generations and subsequently in embryogenesis (*N_e_Emb*, in comparison to *N_e_Oog*). Additionally, five further tissues (gills, heart, muscle, liver and gonads) were sampled from two founder females (214, 263) to investigate whether heteroplasmy levels are constant within different tissues taken from the same individual.

### Pyrosequencing

Haplotype frequencies were determined using quantitative pyrosequencing, undertaken at the National Genetics Reference Laboratory (Wessex, UK) at the Salisbury District Hospital. PCR products were generated in a 50 µl reaction volume with 15 pmol of forward and reverse PCR primers (for sequence details see supporting information), 0.2 mM dNTPs (Promega, Madison, USA), 1.5 mM MgCl_2_, 1× Buffer II (Applied Biosystems), 1 U AmpliTaq Gold (Applied Biosystems, Foster City, USA) using approximately 10 ng genomic DNA. PCR conditions for all reactions were 94°C for 7 min; 40 cycles with denaturation at 94°C for 30 sec, annealing at 57°C for 30 sec and elongation at 72°C for 30 sec; 1 cycle at 72°C for 7 min; and a final hold at 15°C.

Single-stranded biotinylated PCR products were prepared for sequencing using the Pyrosequencing™ Vacuum Prep Tool (Qiagen, Hilden, Germany). Three microliter of Streptavidin Sepharose™ HP (Amersham, Little Chalfont, UK) was added to 37 µl Binding buffer (10 mM Tris-HCl pH 7.6, 2 M NaCl, 1 mM EDTA, 0.1% Tween 20) and mixed with 20 µl PCR product and 20 µl high purity water for 10 min at room temperature using a thermal shaker. The beads containing the immobilized templates were captured onto the filter probes after applying the vacuum, and then washed with 70% ethanol for 5 sec, denaturation solution (0.2 M NaOH) for 5 sec and washing buffer (10 mM Tris-Acetate pH 7.6) for 5 sec. The vacuum was then released and the beads released into a PSQ 96 Plate Low, containing 45 µl annealing buffer (20 mM Tris-Acetate, 2 mM MgAc_2_ pH 7.6) and 0.3 µM sequencing primer (Table S1). Samples were heated to 80°C for 2 min and allowed to cool to room temperature.

Pyrosequencing reactions were performed on a PSQ™ 96MA System according to the manufacturer's instructions, using the PSQ 96 SNP Reagent Kit and analysed using the in-built Allele Frequency Quantification (AQ) function in the SNP Software. For nucleotide dispensation, see supporting information. To determine the measurement error of this method, all females and a randomly chosen subset of oocytes and offspring were subject to repeat measurements. Repeat measurements were carried out on the same DNA extraction.

### Overview of Inheritance Model

In this analysis, we assume there are at most two mitochondrial alleles present in any individual and label them ‘A’ and ‘B’ arbitrarily. Then the heteroplasmy ratio *h* of an individual is the fraction of its genomes belonging to allele A. When comparing the heteroplasmy ratios of two individuals with the same two alleles, we use the same labeling for both of them.

Perhaps the simplest model of the inheritance of mitochondrial heteroplasmy is that each offspring samples a small number *N* of mitochondrial genomes at random from its mother (single-sampling binominal model), defining the offspring's heteroplasmy ratio by the proportions of both samples in this sample [43]. The probability of each sampled genome being allele A is the mother's heteroplasmy ratio *h*. If *h′* is the offspring's heteroplasmy ratio, then *Nh′* has a binomial distribution, so the distribution of *h′* has mean *h*, variance 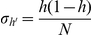, and for large enough *N* (approximately *N>30*) is well approximated by a Gaussian.

If the germline sequence of cells over a full generation had a single bottleneck cell with *N* mitochondrial genomes (all other cells having a much larger number), then our simple model would be close to reality. In reality the situation is likely more complex than this, and the population genetic model assumes the mitochondrial bottleneck is the effect of repeated sampling and partitioning of mitochondrial genomes during all successive binary cell divisions forming the female germ line [8]. In this case the variance in *h′* will be the sum of variances from each bottleneck cell, 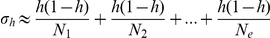 (making the approxinmation that *h(1−h)* does not vary greatly over cell generations) where 
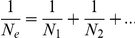
. If 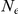 is large enough, the distribution is still well approximated by a Gaussian. Should *h* drift close to 0 or 1during this process, the distribution derived by Wonnapinij et al. [44] exceeds the performance of our simple Gaussian approximation, but the sensitivity limitations of our measurements have the side effect of keeping our estimates well within the zone where the Gaussian approximation is valid.

We therefore model the inheritance of mitochondrial heteroplasmy as follows: a mother with heteroplasmy ratio *h* has offspring with heteroplasmy ratios normally distributed with mean *h* and variance 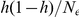. 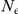 is a property of the population (species) - all mothers have the same value. Our model is only valid if 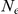 is sufficiently large – the predicted probability of *h′* lying outside the range 0 to 1 increases as *N_e_* decreases and as *h* tends to 0 or 1. For *h* values typical for our data, the approximation will not break down in this way until about *N_e_*<4.

Many biological mechanisms will result in such a distribution - *e.g.*, a bottleneck spread over several cell generations, selection of clusters of mitochondrial genomes, genome reproduction from a subpopulation of the genomes in a single cell [6] or the drift in heteroplasmy in a single cell with lifetime longer than the lifetime of the mitochondrial genomes it contains. Our model and analysis are blind to these details. The parameter which we measure – 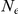 – is a lower bound on the true bottleneck genome number. Its relationship to the true bottleneck genome number will depend on the actual biological mechanisms at play. Our model does not account for the possibility of a bottleneck between the germline cells and the somatic cells sampled from the mother. We would require data from an additional generation of fish to be able to measure this effect. This model is developed in greater detail in Hendy *et al.* [45] and is applied to Adélie penguins in Miller *et al.* [46]. For detailed analyses and calculations, see supporting information (Text S1, S2, S3).

## Results

Two discrete heteroplasmic sites were investigated in this work. Both sites confer to synonymous changes and are located in mitochondrial gene *mt-nd1*, at nucleotide positions 4149 and 4316 (NCBI:NC_002980). An A/G polymorphism (A = wildtype) was found at position 4149 (A4149G), and a C/T polymorphism (T = wildtype) at position 4316 (T4316C). Heteroplasmy levels in the somatic tissue (fin clips) of five female fish, and multiple oocytes (N = 13 to 28) and offspring (N = 20) of each female were examined. Two females were heteroplasmic for nucleotide position 4316 (families 272, 357), and three were heteroplasmic for position 4149 (individual 214, 256, 263). Heteroplasmic offspring were generated by crossing homoplasmic males with heteroplasmic females and the haplotype frequencies of the resulting offspring were determined using quantitative pyrosequencing [47].

Mean levels of heteroplasmy for each developmental stage, expressed as haplotype frequencies within an individual, are shown in Table 1 (see Table S2 for raw data). The variation about these means for the oocyte and offspring samples, and the experimental error, is used to estimate *N_e_* values with our mathematical model. In Table 2, the level of measurement error obtained with the pyrosequencing approach to allelic quantization is indicated by the mean standard errors, and was calculated formally using our model.

**Table 1:** Summary of heteroplasmy levels for each family [% mutant allele].

Family	214	256	263	272	357
Heteroplasmy	A4149G	A4149G	A4149G	T4316C	T4316C
**Mother mean**	63.3	32.4	67.7	20.3	28.1
**n**	13	13	13	12	10
**C of V (%)**	1.6	4.0	1.1	15.1	8.6
**Oocyte mean**	63.8	37.4	67.6	20.9	26.3
**N**	28	23	13	20	17
**SD**	5.1	4.3	4	4.4	5.3
**C of V (%)**	8.0	11.6	6.0	21.1	20.0
**Offspring mean**	65.0	36.2	69.9	19.8	31.6
**N**	20	20	20	20	20
**SD**	6.4	4.4	3.6	4.5	4.4
**C of V (%)**	9.8	12.1	5.2	22.8	13.9

Values represent percentage of the G or C allele, respectively. n: number of repeat measurements from one individual, N: number of individuals from which measurements taken, SD: standard deviation; C of V: coefficient of variance.

**Table 2:** Variation in heteroplasmy levels between measurements at each developmental stage of randomly chosen oocyte and offspring samples and of all females to determine the measurement error.

	n	N	Mean SE
**Mother**	10 to 13	5	0.50
**Oocyte**	3	31	0.88
**Offspring**	3	18	0.58

n: number of repeat measurements per sample, N: total number of individuals from which repeat measurements were taken, mean SE: mean standard error across all individuals.

To determine whether fin clips are a good representation of heteroplasmy levels in all cell lines of mother fish, we measured heteroplasmy levels in various tissues of two female founder fish (Table 3). One-sample t-tests were performed, comparing heteroplasmy levels from fin clips with those obtained from five other tissue types for two family lines, 214 and 263. Of the ten p-values obtained, two were less than the cut-off value of 0.05, with no obvious trend between tissue types. Some chance fluctuation in heteroplasmy levels between tissues is expected due to the random segregation of mtDNA, and may explain the two significant p-values. Since fin clip measurements were not significantly different between 4 out of 5 tissues in both family lines, we deemed our estimation of heteroplasmy in the mother fish a good approximation of what existed in the primordial germ cells (Table 3).

**Table 3:** Triplicate measurements of heteroplasmy levels G4149A in various tissues of mothers 214 and 263 [% mutant allele].

	214			263		
	Mean	SE	p	Mean	SE	p
**Gills**	64.0	0.7	0.39	68.1	0.3	0.33
**Gonads**	65.6	0.4	0.03	69.7	0.6	0.08
**Heart**	63.9	0.3	0.21	69.2	0.7	0.15
**Liver**	63.9	0.5	0.33	69.7	0.2	0.01
**Muscle**	65.5	0.5	0.05	67.3	0.5	0.50

Values represent percentage of the G allele. SE: standard error. P-values from one sample t-tests with fin clip heteroplasmy measurements are shown.

**Figure 2:**
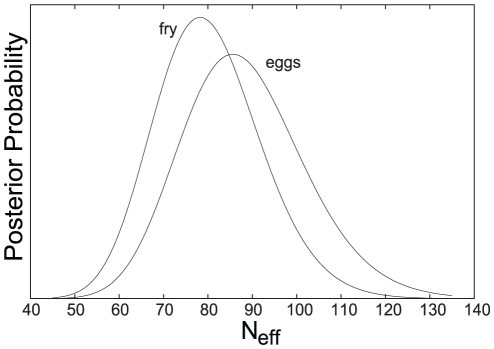
Posterior distributions on *N_e_*, the effective bottleneck number of mitochondrial genomes per cell.

**Figure 3:**
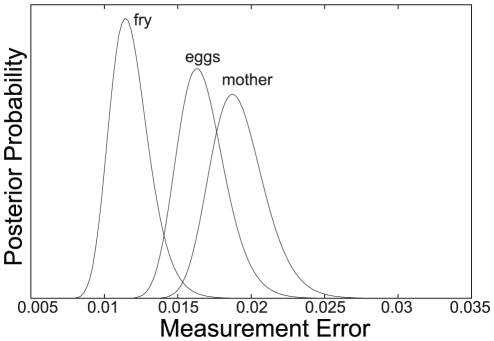
Posterior distribution on heteroplasmy measurement error.

The heteroplasmy levels observed in offspring are not significantly different from those observed in oocytes (p = 0.33). Consequently, *N_e_* for offspring is of similar size to the *N_e_* estimated for eggs, indicating no reduction in mitochondrial genome number between these two stages (Figure 2): Analyzing the data from the eggs with our model gives a posterior distribution on *N_e_* with mean 88.3, median 87.4, mode 85.6, 95% confidence interval 63.7 to 118.4. For the offspring data, the posterior distribution on *N_e_* has mean 80.3, median 79.6, mode 78.2, 95% confidence interval 58.7 to 105.8. The posterior distributions on measurement errors are plotted in Figure 3. The mean (and 95% CIs) are 1.92% (1.59%–2.32%) for mothers, 1.67% (1.38%–2.03%) for eggs, and 1.18% (0.95%–1.49%) for offspring. Our lower *N_e_* estimate of 80.3 is approximately 4.0×10^7^-fold less than mtDNA copy number in mature salmon oocytes, estimated to harbor some 3.2×10^9^ mtDNA molecules [39].

## Discussion

We draw two major conclusions from our work. Firstly, by observing changes in heteroplasmy levels from mothers to oocytes and offspring, we can confirm that a mitochondrial genetic bottleneck, or bottleneck effect, exists in chinook salmon during early developmental stages. Thus, salmon appear congruent with an array of other taxonomic groups [7], [8], [12], [19], [20], [30], [31], [32], [34], [35], [36]. Secondly, the similarity between *N_e_Oog* (88.3) and *N_e_Emb* (80.3), as found here, indicates that the main mitochondrial genetic bottleneck occurs during oogenesis in salmon. Although we found *N_e_Oog* to be slightly higher than *N_e_Emb*, this difference was not significantly different. As estimates of *N_e_* are directly correlated to measurement error, the greater value of *N_e_* for oogenesis may be due to a larger measurement error for oocytes (1.67%), compared to that for F1 offspring (1.18%). Alternatively, random sample choice could have led to the chance selection of oocyte samples with less variation in heteroplasmy measurements, leading to a higher estimate of *N_e_Oog*. A third possibility is that a further, non-significant, reduction in effective mtDNA copy number occurs during embryogenesis.

Our results tie in with those of earlier studies which proposed that segregation is likely to occur prior to the differentiation of the primary oocyte population during oogenesis in mouse [8], [21]. Possible mechanisms underlying heteroplasmy shifts in oocytes have been proposed and include relaxed replication of mtDNA, and random partitioning of mitochondria [8], [48], which typically depend on vast cell proliferation and mtDNA replication, two processes that can be observed during germ line development [19], [49].

Previous studies suggest that the size of the intergenerational mtDNA bottleneck is unexpectedly similar across a wide range of different taxa, spanning invertebrates to vertebrates, supporting the idea of a conserved mechanism across taxa [7], [8], [12], [31], [34], [36]. Our estimates of *N_e_Oog* and *N_e_Emb* for chinook salmon are in concordance with those of mammals [8], [20] and crickets [31], and within the same order of magnitude as fruit flies [12]; despite the 10,000-fold higher mtDNA content of chinook salmon versus mammalian oocytes, and differences in cleavage patterns (rotational holoblastic in mammals vs. discoidal meroblastic in teleosts) [34], [39], [50]. Further, the mechanism in salmon demonstrates striking similarities to those found in mice, according to two studies, in that segregation of heteroplasmy can be accounted for during oogenesis [8], [21]. Thus, our findings strongly indicate that mechanisms of mitochondrial inheritance may be conserved and of a comparable nature among divergent taxa.

The intergenerational transmission of mitochondrial heteroplasmy has many important biological implications. First, the transmission of heteroplasmy has direct impact on the inheritance of mitochondrial diseases [1], [51], [52], [53] - most of which are observed in a heteroplasmic form and expressed when the deleterious allele exceeds a certain threshold [1], [4]. Second, heteroplasmy may also create some ambiguity in phylogenetic and network interpretations of population data of mtDNA [2]. Third, heteroplasmy will create the possibility for intermolecular recombination [2], [3], which might further affect the evolution of the mtDNA molecule and thus evolutionary analysis based on this molecule [54], but also may enable the molecule to escape the mutational meltdown expected if it were solely inherited in a clonal fashion [28], [55]. On the other hand, the inclusion of knowledge of the frequency and stability of mtDNA heteroplasmy would increase the level of molecular information available from, and may improve the resolution of, mtDNA focused analyses in evolutionary, forensic and medical science [2].

For example, heteroplasmic states that are stably inherited for significant periods may provide useful additional information for defining haplotypes, and resolving further the relationships among individuals at a population level [2]. Thus far such an approach has been used rarely [2], [16], but there is potential for this to increase if we better understand the mtDNA bottleneck and can therefore predict how long such mutations might persist. A simple mutation drift model predicts that a mtDNA bottleneck of N_e_ = 100 leads to a predicted time to fixation for a neutral, mitochondrial, heteroplasmic variant of approximately 200 generations [2]; long enough to impact significantly on population genetic interpretations.

In this study, we have demonstrated the existence of a mtDNA bottleneck in a teleost, the first non-mammalian vertebrate to be examined. Despite fundamental differences in physiology and developmental cleavage pattern, the number of segregating units between generations appears remarkably similar to that found in other species, including mouse. This finding suggests the mitochondrial bottleneck might be conserved among divergent taxa. However, across the animal kingdom, our knowledge of the mechanisms underlying mtDNA inheritance is far from complete, and many more studies of this nature are required to further understand this important evolutionary process, and thus capture the full extent of the additional value that an understanding of heteroplasmy may bring to the life sciences.

## Supporting Information

Text S1 Detailed mathematical analysis.
(DOC)

Text S2 Mathematica notebook (Mathematica file).
(NB) Click here for additional data file.

Text S3 Mathematica notebook (.pdf version).
(PDF)

Table S1 Primer and target sequences and dispensation order for pyrosequencing to determine allele frequencies for two heteroplasmic sites.
(DOC)

Table S2 Raw data for a.mothers (fin clips, additional repeat measurements (1–10) to infer measurement error in founder females), b.oocytes and c.offspring. Frequencies are expressed as x(10^1^). More than one number in one cell indicates repeat measurements for that sample.
(DOC)
