# Supplementary material for: The Strength and Timing of the Mitochondrial Bottleneck in Salmon Suggests a Conserved Mechanism in Vertebrates
Source: PLoS One. 2011 May 31;6(5):e20522. doi: 10.1371/journal.pone.0020522 (PMC3105079; doi:10.1371/journal.pone.0020522)
Supplement: Text S1 — Detailed mathematical analysis. (DOC) [file pone.0020522.s001.doc]

**Mathematical Analysis**

**Overview of Analysis Method**: We seek to determine
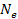
from the variability in heteroplasmy ratio between mother and offspring. We partition our data into measurements from eggs and measurements from fry, and determine
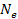
 separately for each partition, to see whether there is a mitochondrial genome bottleneck between egg and fry.

Consider the case where we have a number of independent mother/child pairs, with a measurement of heteroplasmy from each. The mother-to-offspring variance in measured heteroplasmy is due to the actual mother-to-offspring heteroplasmy variance
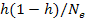
 plus measurement variances (due to experimental uncertainty). It is therefore necessary to estimate the measurement variances as 'nuisance parameters' in order to find
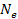
.

We measure the heteroplasmy ratio multiple times for some of our individuals to estimate the measurement uncertainty, and use a Bayesian likelihood analysis to get a posterior probability distribution on
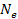
.

**Detailed Analysis**: Previously we considered a collection of independent mother-offspring measurement pairs. Our actual situation is more complex. For the mother (and sometimes the offspring) we have multiple measurements and, for each mother, we have multiple offspring.

Let
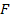
be the number of families and
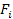
the number of children measured from family
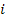
 (
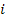
in the range 1 to
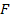
) Let
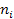
be the number of heteroplasmy measurements taken from mother
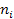
 , and
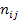
be the number of measurements from child
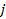
of family
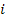
(
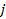
in the range 1 to
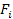
). The heteroplasmy measurements are
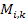
from the mothers (from 1 to
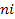
) and
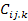
from offspring (
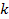
 from 1 to
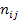
).

We assume that within each class of sampled organisms (mothers, eggs and fry) the measurement errors are uniform, but the errors may differ between classes. For the mothers, the likelihood of the measurement error
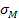
 is
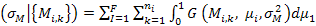
 (eq 1)

where
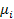
is a possible true heteroplasmy value for mother *i* and
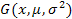
 is the probability density function (PDF) of a Gaussian distribution of mean
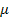
 and variance
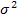
. Then, the posterior distribution on
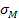
is
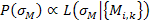
 (eq 2)

(assuming a uniform prior on
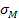
, and the constant of proportionality is found by the requirement that the integral of
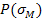
 equals one). The posterior distribution on the offspring measurement error
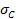
is calculated in the same way. We calculate the distributions by numerical integration.

Consider a mother with true heteroplamsy *m* and offspring with true heteroplasmy *c*. We measure the heteroplasmy of the mother
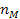
times, then the mean of these measurements
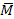
 estimates *m* with variance
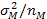
, and similarly we have
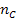
measurements from the offspring with mean
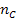
. Assuming independence,
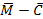
 is Gaussian with mean zero and variance
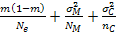
 . Then the likelihood of
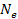
,
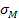
and
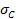
is


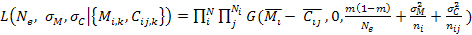
 (eq 3)

and
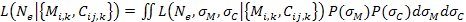
 (eq 4)

where
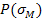
 and
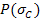
 are estimated from equation 2.

Finally, the posterior distribution on
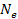
is
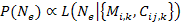
 (eq 5)

where we have assumed a uniform prior on
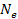
.

**Analysis of Results**: The analysis was done in *Mathematica* using numerical integrations, and using interpolating functions to approximate
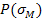
,
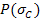
 and
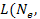

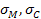
. A *Mathematica* notebook file is included in the supplementary material, along with a PDF version. The notebook also includes a Monte Carlo simulation to test the accuracy of the analysis.
