# Supplementary material for: The Strength and Timing of the Mitochondrial Bottleneck in Salmon Suggests a Conserved Mechanism in Vertebrates
Source: PLoS One. 2011 May 31;6(5):e20522. doi: 10.1371/journal.pone.0020522 (PMC3105079; doi:10.1371/journal.pone.0020522)
Supplement: Text S3 — Mathematica notebook (.pdf version). (PDF) [file pone.0020522.s003.pdf]

Data analysis for the paper "An Extensive Mitochondrial Bottleneck Occurs During Oogenesis in a Non-Mammalian Vertebrate" by Jonci N Wolff, Daniel J
White, Michael Woodhams, Helen E White and Neil J Gemmell.

The data analysis has been done in *Mathematica*. As this is an expensive commercial package, many will not have access to it or be familiar with it. I have assumed the reader is familiar with several computer programming languages, and I give additional explainations where such experience does not suffice to understand *Mathematica*'s syntax. (*Mathematica* can be used like a procedural language, but it is more friendly to being used in functional and logical (e.g. Prolog) programming idioms.)

Predefine some variables to avoid warning messages when variable names are similar:

```
In[1]:= Off[General::spell, General::spell1];
        {rawData, mData, meData, eData, fData, mfData, efData, normal, norm,
          σPDFmData, σPDFeData, σPDFfData, σPDFmeData, σPDFmfData, σLogPDFmData,
          σLogPDFmeData, σLogPDFmfData, σLogPDFefData, σLogPDFeData, σLogPDFfData,
          σLogPDFtmData, σPDFtmData, σLogPDFtmeData, σPDFtmeData, σLogPDFtmfData,
          σPDFtmfData, tmData, mean, median, mode, var, varM, varC, vard, nMin, nMax,
          grid, logInterp3Dtmef, logInterp3Dtme, logInterp3Dtmf, interp1Dtmef,
          interp1Dtme, interp1Dtmf, logInterp3Dme, interp1Dme, logInterp3Dmf,
          interp1Dmf, mean, meanM, meanC, tmeData, tmfData, xMin, xMax, yMin, yMax};
        On[General::spell, General::spell1];
```

Turn off some numerical warnings which will otherwise be obtrusive:

```
In[4]:= Off[NIntegrate::ncvb, NIntegrate::slwcon];
```

This is the raw data. Each individual has a list of its name followed by its heteroplasmy measurements in parts-per-thousand. Each family is a list of individual lists, with the mother coming first. The whole stricture is a list of families.

```
In[5]:= rawData =
          {{{"m214", 634, 654, 637, 627, 632, 616, 630, 638, 645, 639, 616, 636, 630}, {"e1"},
            {"e2"}, {"e3", 597}, {"e4", 630}, {"e5", 654}, {"e6", 597}, {"e7", 578, 611},
            {"e8", 690}, {"e9", 500}, {"e10", 608, 597, 589}, {"e11", 574, 571, 578},
            {"e12", 669, 637, 654}, {"e13", 667, 653, 636}, {"e14", 770, 758, 772},
            {"e15", 638, 628, 605}, {"e16", 680, 648, 649}, {"e17", 627, 641, 625},
            {"e18", 566, 564, 563}, {"e19", 638, 624, 616}, {"e20", 666, 664, 674},
            {"e21", 651, 629, 615}, {"e22", 686, 660, 685}, {"e23", 686, 670, 656},
            {"e24", 710, 679, 700}, {"e25", 656, 656, 640}, {"e26", 725, 704, 708},
            {"e27", 621, 633, 631}, {"e28", 672, 653, 649}, {"e29", 647, 649, 633},
            {"e30", 634, 612, 621}, {"f1 ", 634, 654, 637}, {"f2 ", 649}, {"f3 ", 736},
            {"f4 ", 736, 744, 752}, {"f5 ", 749}, {"f6 ", 640}, {"f7 ", 628, 635, 641},
            {"f8 ", 618}, {"f9 ", 597}, {"f10 ", 640}, {"f11", 606}, {"f12", 654, 668, 642},
            {"f13", 600, 585, 576}, {"f14", 609, 610, 588}, {"f15", 690, 684, 690},
            {"f16", 637, 641, 639}, {"f17", 717}, {"f18", 485}, {"f19", 692}, {"f20", 703}},
           {{"m256", 330, 318, 326, 333, 339, 302, 338, 315, 316, 333, 308, 341, 310},
            {"e1"}, {"e2", 374}, {"e3"}, {"e4"}, {"e5"}, {"e6"}, {"e7"}, {"e8", 357, 323},
            {"e9", 329}, {"e10"}, {"e11", 332}, {"e12", 394}, {"e13", 337},
            {"e14", 409}, {"e15", 434}, {"e16", 401}, {"e17", 395}, {"e18", 341},
            {"e19", 358}, {"e20", 461}, {"e21", 274}, {"e22", 340}, {"e23", 363},
            {"e24", 408}, {"e25", 397}, {"e26", 340}, {"e27", 395}, {"e28", 339},
            {"e29", 383}, {"e30", 441}, {"f1 ", 397, 399, 404}, {"f2 ", 287},
            {"f3 ", 332}, {"f4 ", 313, 297, 344}, {"f5 ", 334}, {"f6 ", 333},
```

```
    {"f7 ", 338}, {"f8 ", 311}, {"f9 ", 329, 344, 327}, {"f10 ", 413},
    {"f11", 430}, {"f12", 353}, {"f13", 445}, {"f14", 340}, {"f15", 355},
    {"f16", 362}, {"f17", 428}, {"f18", 368}, {"f19", 351}, {"f20", 403}},
   {{"m263", 672, 670, 678, 668, 672, 695, 679, 679, 669, 683, 676, 676, 681},
    {"e1", 591}, {"e2", 696}, {"e3"}, {"e4"}, {"e5", 630}, {"e6"}, {"e7"},
    {"e8"}, {"e9"}, {"e10"}, {"e11", 699}, {"e12", 675}, {"e13", 664},
    {"e14", 716}, {"e15", 659}, {"e16", 643}, {"e17", 675}, {"e18", 736},
    {"e19", 726}, {"e20", 676}, {"e21"}, {"e22"}, {"e23"}, {"e24"},
    {"e25"}, {"e26"}, {"e27"}, {"e28"}, {"e29"}, {"e30"}, {"f1 ", 765},
    {"f2 ", 698, 697, 700}, {"f3 ", 648}, {"f4 ", 695}, {"f5 ", 669}, {"f6 ", 729},
    {"f7 ", 652, 637, 658}, {"f8 ", 694}, {"f9 ", 642}, {"f10 ", 708, 681, 702},
    {"f11", 731}, {"f12", 720}, {"f13", 660}, {"f14", 724}, {"f15", 712},
    {"f16", 763}, {"f17", 658}, {"f18", 722}, {"f19", 685}, {"f20", 714}},
   {{"m272", 246, 237, 259, 200, 199, 194, 196, 210, 179, 156, 192, 169},
    {"e1", 323}, {"e2", 283, 253, 255}, {"e3", 222}, {"e4", 228},
    {"e5", 236}, {"e6", 199}, {"e7", 273, 258, 240}, {"e8", 155}, {"e9", 182},
    {"e10", 197, 147, 171}, {"e11", 239}, {"e12", 150}, {"e13", 151},
    {"e14", 203}, {"e15", 228}, {"e16", 175}, {"e17", 208}, {"e18", 236},
    {"e19", 183}, {"e20", 169}, {"e21"}, {"e22"}, {"e23"}, {"e24"}, {"e25"},
    {"e26"}, {"e27"}, {"e28"}, {"e29"}, {"e30"}, {"f1 ", 246, 237, 259},
    {"f2 ", 276}, {"f3 ", 273}, {"f4 ", 226, 220, 234}, {"f5 ", 190}, {"f6 ", 152},
    {"f7 ", 181, 188, 189}, {"f8 ", 243}, {"f9 ", 173}, {"f10 ", 254},
    {"f11", 154}, {"f12", 166}, {"f13", 144}, {"f14", 149}, {"f15", 180},
    {"f16", 242}, {"f17", 152}, {"f18", 201}, {"f19", 212}, {"f20", 139}},
   {{"m357", 303, 316, 292, 254, 298, 268, 283, 294, 243, 256}, {"e1", 300},
    {"e2", 263}, {"e3", 343}, {"e4"}, {"e5", 340, 314, 345}, {"e6"}, {"e7", 284},
    {"e8", 325, 259, 239}, {"e9"}, {"e10", 236}, {"e11", 254}, {"e12", 277},
    {"e13", 343}, {"e14", 153}, {"e15", 240}, {"e16", 211}, {"e17", 216},
    {"e18", 224}, {"e19", 310}, {"e20", 214}, {"e21"}, {"e22"}, {"e23"}, {"e24"},
    {"e25"}, {"e26"}, {"e27"}, {"e28"}, {"e29"}, {"e30"}, {"f1 ", 248, 279, 278},
    {"f2 ", 408}, {"f3 ", 278}, {"f4 ", 288, 269, 278}, {"f5 ", 310}, {"f6 ", 334},
    {"f7 ", 275}, {"f8 ", 369}, {"f9 ", 330, 296, 303}, {"f10 ", 267},
    {"f11", 339}, {"f12", 294}, {"f13", 288}, {"f14", 270}, {"f15", 294},
    {"f16", 347}, {"f17", 359}, {"f18", 357}, {"f19", 278}, {"f20", 389}}};
```

Now we strip of the names to leave just numbers in 'data', convert parts-per-thousand to a fraction, and make some subsets: mData is mothers only, eData eggs only, fData fry only, meData mothers and eggs, mfData mothers and fry.

```
In[6]:= data = Map[Rest, rawData, {2}] / 1000.;
        mData = data〚All, {1}〛;
        eData = data〚All, Range[2, 31]〛;
        fData = data〚All, Range[32, 51]〛;
        meData = data〚All, Range[31]〛;
        mfData = data〚All, Join[{1}, Range[32, 51]]〛;
```

For example, here's the data for family 1, mother and eggs:

```
In[12]:= meData⟦1⟧

Out[12]= {{0.634, 0.654, 0.637, 0.627, 0.632, 0.616, 0.63, 0.638, 0.645, 0.639, 0.616, 0.636,
          0.63}, {}, {}, {0.597}, {0.63}, {0.654}, {0.597}, {0.578, 0.611}, {0.69},
         {0.5}, {0.608, 0.597, 0.589}, {0.574, 0.571, 0.578}, {0.669, 0.637, 0.654},
         {0.667, 0.653, 0.636}, {0.77, 0.758, 0.772}, {0.638, 0.628, 0.605},
         {0.68, 0.648, 0.649}, {0.627, 0.641, 0.625}, {0.566, 0.564, 0.563},
         {0.638, 0.624, 0.616}, {0.666, 0.664, 0.674}, {0.651, 0.629, 0.615},
         {0.686, 0.66, 0.685}, {0.686, 0.67, 0.656}, {0.71, 0.679, 0.7},
         {0.656, 0.656, 0.64}, {0.725, 0.704, 0.708}, {0.621, 0.633, 0.631},
         {0.672, 0.653, 0.649}, {0.647, 0.649, 0.633}, {0.634, 0.612, 0.621}}
```

This is the Gaussian/Normal distribution function

```
In[13]:= normal[x_, μ_, var_] := 1 / (Sqrt[var 2 Pi]) * Exp[- (x - μ) ^2 / (2 var)];
```

Now calculate the likelihood of a given measurement error. It is possible to do the following analytically, but just throwing numerical integrations at the problem is simpler to understand.

'likelihood$\mu\sigma$' is the likelihood of the data (a list of numbers) for the given μ and σ. (We apply the 'normal' function to each element of 'data', then multiply the results.

```
In[14]:= likelihoodμσ[μ_, σ_, data_List] := Times @@ Map[normal[#, μ, σ^2] &, data];
```

This function finds the log likelihood for a value of σ given a list of measurements from a single individual. We eliminate μ by integrating over it. (In principle we integrate from $-\infty$ to $+\infty$, but I omit the range where the function is known to be very small.)

```
In[15]:= logLikelihoodσ1[σ_, data_List] := If[Length[data] < 2, 0, Log[
            NIntegrate[likelihoodμσ[μ, σ, data], {μ, Min[data] - 5 * σ, Max[data] + 5 * σ}]]];
```

Now 'array' is a list of lists (data from a single family), and each list (data from an individual) has the same measurement error σ but they all have different unknown means. The log likelihood of σ is just sum of the log likelihoods for each individual:

```
In[16]:= logLikelihoodσ2[σ_, array_List] := Plus @@ Map[logLikelihoodσ1[σ, #] &, array];
```

Finally logLikelihoodσ3 takes a list of data from all families and applies logLikelihoodσ2 to each family and adds the results.

```
In[17]:= logLikelihoodσ3[σ_, array_List] := Plus @@ Map[logLikelihoodσ2[σ, #] &, array];
```

As an illustration, here is a plot of the log likelihood if we combine all the data (i.e. assume all measurements have the same error):

```
In[18]:= Plot[logLikelihoodσ3[σ, data], {σ, 0.01, 0.05}];
```

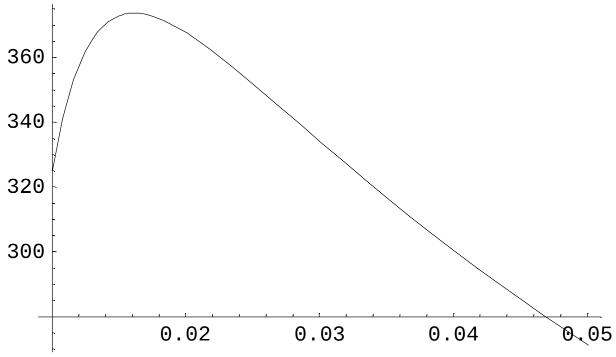

This is a bit slow to calculate, as it requires integration, so we evaluate it on some grid points and create an interpolating function over those grid points to save time.

This procedure takes an input function and returns two approximating functions: one approximates the input function, the other is the exponential of the input function normalized to integate to one. (I.e. if the input function is a log likelihood, the second approximating function is the corresponding posterior probability, assuming a flat prior.)

```
In[19]:= interpolationFromLogLikelihood[f_, min_, max_] := Module[
           {data, logInterp, norm, dataMax},
           data = Table[{x, f[x]}, {x, min, max, (max - min) / 50}];
           logInterp = Interpolation[data];
           (* Scale data to avoid over/underflow *)
           dataMax = Max[data[[All, 2]]];
           norm = NIntegrate[Exp[logInterp[x] - dataMax], {x, min, max}];
           linInterp = Interpolation[Map[{#[[1]], Exp[#[[2]] - dataMax] / norm} &, data]];
           Return[{logInterp, linInterp}];
          ];
```

And we create interpolating functions for our various subsets of the data. (This takes a few seconds to calculate. The warning message is not significant.)

```
In[20]:= minσ = 0.005; maxσ = 0.035;
         {σLogPDFallData, σPDFallData } =
          interpolationFromLogLikelihood[logLikelihoodσ3[#, data] &, minσ, maxσ];
         {σLogPDFmData, σPDFmData} = interpolationFromLogLikelihood[
           logLikelihoodσ3[#, mData] &, minσ, maxσ];
         {σLogPDFeData, σPDFeData} = interpolationFromLogLikelihood[
           logLikelihoodσ3[#, eData] &, minσ, maxσ];
         {σLogPDFfData, σPDFfData} = interpolationFromLogLikelihood[
           logLikelihoodσ3[#, fData] &, minσ, maxσ];
         {σLogPDFmeData, σPDFmeData} = interpolationFromLogLikelihood[
           logLikelihoodσ3[#, meData] &, minσ, maxσ];
         {σLogPDFmfData, σPDFmfData} = interpolationFromLogLikelihood[
           logLikelihoodσ3[#, mfData] &, minσ, maxσ];
```

For example, for all the data lumped together:

```
In[27]:= Plot[σLogPDFallData[x], {x, 0.005, 0.035}];
         Plot[σPDFallData[x], {x, 0.005, 0.035}, PlotRange → All];
```

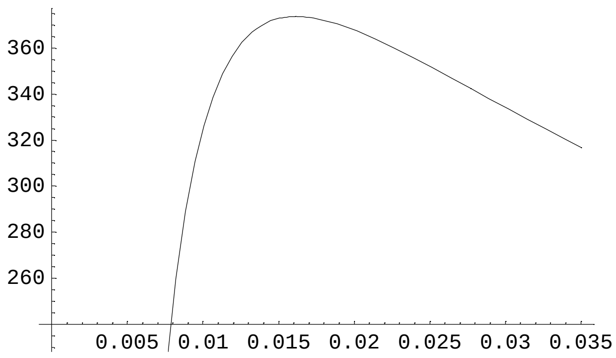

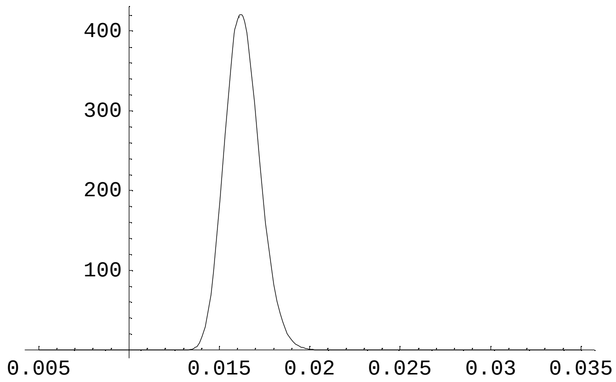

Now we can see whether the measurement errors for the different types of sample are the same:

```
In[29]:= graph = Plot[{σPDFmData[σ], σPDFeData[σ], σPDFfData[σ]}, {σ, minσ, maxσ},
           PlotRange → {{0, maxσ}, All}, Axes → {True, False}, AxesLabel → {"P(σ)", None}];
```

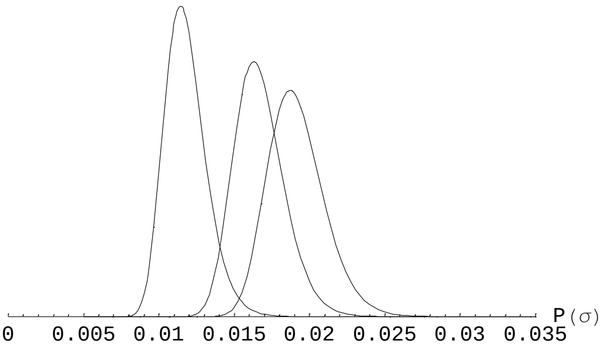

and we see that they are not (Peaks are fry, eggs, mothers from left to right.)

This function finds mean, median, mode, 95% confidence intervals on a probability density function. (Confidence interval is from 2.5th percentile to 97.5th percentile.)

```
In[30]:= stats[f_, min_, max_] := Module[{answer, x, μ},
           Off[NIntegrate::nlim,
            NIntegrate::slwcon, NIntegrate::ncvb, FindMaximum::lstol];
           μ = NIntegrate[x f[x], {x, min, max}];
           answer = {{mean → μ},
             FindRoot[NIntegrate[f[x], {x, min, median}] == 0.5, {median, μ, min, max}],
             FindMaximum[f[mode], {mode, μ, min, max}][[2]],
             FindRoot[NIntegrate[f[x], {x, min, lower}] == 0.025, {lower, μ, min, max}],
             FindRoot[NIntegrate[f[x], {x, min, upper}] == 1 - 0.025, {upper, μ, min, max}]
            };
           On[NIntegrate::nlim,
            NIntegrate::slwcon, NIntegrate::ncvb, FindMaximum::lstol];
           answer
          ];
         stats[f_InterpolatingFunction] := stats[f, f[[1, 1, 1]], f[[1, 1, 2]]];
```

And here are those distribution statistics for mothers, eggs and fry:

```
In[32]:= stats[σPDFmData]
         stats[σPDFeData]
         stats[σPDFfData]

Out[32]= {{mean → 0.019152}, {median → 0.0190061},
          {mode → 0.0187362}, {lower → 0.0159259}, {upper → 0.0232228}}

Out[33]= {{mean → 0.0166863}, {median → 0.0165551},
          {mode → 0.0163152}, {lower → 0.0138276}, {upper → 0.0203106}}

Out[34]= {{mean → 0.0118028}, {median → 0.0116785},
          {mode → 0.0114515}, {lower → 0.00948202}, {upper → 0.0148732}}
```

Now we can move on to estimating N, the bottleneck genome number. (I use 'n' instead of 'N' here as 'N' means something to *Mathematica.*

Notation:
n = bottleneck genome number
m = the mother's actual heteroplasmy ratio
c = the child's actual heteroplasmy ratio
M = mother's measured heteroplasmy ratio (including measurement error)
C = child's measured heteroplasmy ratio
σ_M = measurement error in mother
σ_C= measurement error in child
$\sigma_d{}^2$ = 'drift variance' = $E[(m-c)$^2], measuring the expected intergenerational change in heteroplasmy ratio
σ^2 = 'total variance' = $E[(M-C)$^2], measuring the expected intergenerational change in measured heteroplasmy ratio
$\hat{\sigma}^2$ = estimate of total variance (etc.)

By our model, the child's number of mutant genomes will be drawn from a binomial distribution of size n. From the binomial distribution, the drift variance is $\sigma_d{}^2 = m(1-m)/n$, and except for small n, we can take it to be a normal distribution. Similarly, we assume measurement errors are normally distributed, with zero mean. Given mother and child measurement variances $\sigma_M{}^2$ and $\sigma_C{}^2$, the difference between mother and child measurement is distributed with mean of zero and variance $\sigma^2 = \sigma_M{}^2 + \sigma_C{}^2 + \sigma_d{}^2$(assuming independence between the measurement errors and the drift in genome number) . Hence we can calculate a likelihood of a (M, C) measurement pair for a given n if we know (or assume) values for $\sigma_M{}^2$ and $\sigma_C{}^2$.

This comes from equation 3 in the paper. <Check that equation number has not changed.> It takes the measurements from one mother and the measurements from one child (egg or fry).

```
In[35]:= logLikelihoodDiff[n_, errM_, errC_, Mlist_List, Clist_List] :=
           Module[{meanM, meanC, vard, varM, varC},
            If[Length[Clist] == 0 || Length[Mlist] == 0,
             Return[0],
             meanM = Mean[Mlist];
             meanC = Mean[Clist];
             vard = meanM (1 - meanM) / n;
             varM = errM^2 / Length[Mlist];
             varC = errC^2 / Length[Clist];
             Return[Log[normal[meanM - meanC, 0, varM + varC + vard]]]
            ]
           ];
```

This function adds up the log likelihoods for all children in a single family:

```
In[36]:= logLikelihoodFamily[n_, errM_, errC_, familyData_] :=
           Plus @@ Table[logLikelihoodDiff[n, errM, errC, familyData[[1]], familyData[[i]]],
             {i, 2, Length[familyData]}];
```

And this one adds the log likelihoods for each family:

```
In[37]:= logLikelihood[n_, errM_, errC_, data_] :=
           Plus @@ Map[logLikelihoodFamily[n, errM, errC, #] &, data];
```

For example, here is a plot of the log likelihoods of n if we assume 2% measurement error for both mother and child, and we combine both eggs and fry in a single analysis:

```
In[38]:= nMin = 30; nMax = 150;
         Plot[logLikelihood[n, 0.02, 0.02, data],
           {n, nMin, nMax}, PlotRange → {{0, nMax}, All}];
```

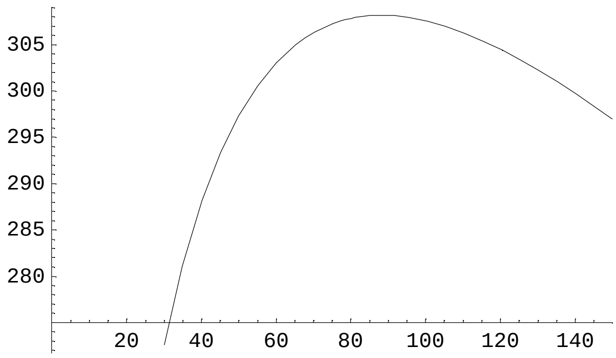

For comparison, see what happens if we assume 1% measurement error instead:

```
In[40]:= Plot[logLikelihood[n, 0.01, 0.01, data],
           {n, nMin, nMax}, PlotRange → {{0, nMax}, All}];
```

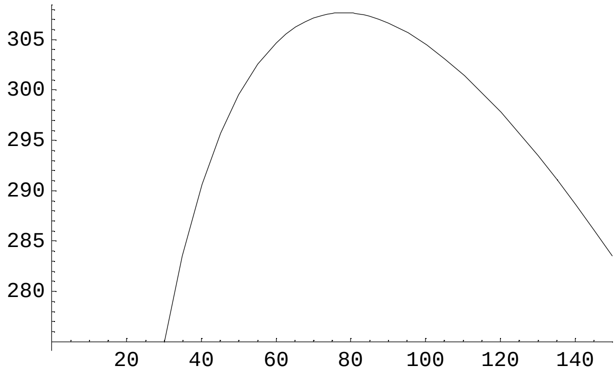

Because less of the difference between mother and child measurements is attributable to measurement error, the genetic drift must be greater hence n must be smaller - the peak shifts from about 85 to about 75.

We now want to eliminate the measurement uncertainties. We form a three dimensional grid over the plausible values of n, σ_M and σ_C. At each point we evaluate the overall likelihood (likelihood of (n, σ_M, σ_C) times likelihood of σ_M times likelihood of σ_C.) The likelihoods of σ_M and σ_C come from the interpolated functions we generated earlier.

This function returns the grid of log likelihoods:

```
In[41]:= logLikelihoodGrid[logErrMInterp_, logErrCInterp_, data_] :=
           Table[{n, errM, errC, logLikelihood[n, errM, errC, data] + logErrMInterp[errM] +
              logErrCInterp[errC]}, {n, nMin, nMax, (nMax - nMin) / 40},
            {errM, minσ, maxσ, (maxσ - minσ) / 40}, {errC, minσ, maxσ, (maxσ - minσ) / 40}];
```

And this function creates a (3D) interpolating function using the grid points:

```
In[42]:= interpolateLogLikelihood[logErrMInterp_, logErrCInterp_, data_] :=
          Module[{grid, peak},
           grid = Flatten[logLikelihoodGrid[logErrMInterp, logErrCInterp, data], 2];
           peak = Max[grid[[All, 4]]];
           grid = Map[# - {0, 0, 0, peak} &, grid];
           Return[Interpolation[grid]];
          ]
```

It takes about 10 minutes to calculate these. (These timings are on an Intel E7300 2.66GHz CPU, a recent mid-range processor.)
This is for the mother-plus-eggs data:

```
In[43]:= Timing[
          logInterp3Dme = interpolateLogLikelihood[σLogPDFmData, σLogPDFeData, meData];]

Out[43]= {461.295 Second, Null}
```

And this is the mother-plus-fry data:

```
In[44]:= Timing[
          logInterp3Dmf = interpolateLogLikelihood[σLogPDFmData, σLogPDFfData, mfData];]

Out[44]= {382.233 Second, Null}
```

And now we can integrate over the nuisance variables: (Warning messages are because the integrand is very small over most of the range.)

```
In[45]:= integrateOverError[n_, logInterp3D_] := NIntegrate[
            Exp[logInterp3D[n, errM, errC]], {errM, minσ, maxσ}, {errC, minσ, maxσ}];

In[46]:= Plot[integrateOverError[n, logInterp3Dme], {n, nMin, nMax}, PlotRange → All]

         NIntegrate::slwcon :
          Numerical integration converging too slowly; suspect one of the following: singularity, value
            of the integration being 0, oscillatory integrand, or insufficient WorkingPrecision. If your
            integrand is oscillatory try using the option Method->Oscillatory in NIntegrate. More…

         NIntegrate::slwcon :
          Numerical integration converging too slowly; suspect one of the following: singularity, value
            of the integration being 0, oscillatory integrand, or insufficient WorkingPrecision. If your
            integrand is oscillatory try using the option Method->Oscillatory in NIntegrate. More…

         NIntegrate::slwcon :
          Numerical integration converging too slowly; suspect one of the following: singularity, value
            of the integration being 0, oscillatory integrand, or insufficient WorkingPrecision. If your
            integrand is oscillatory try using the option Method->Oscillatory in NIntegrate. More…

         General::stop : Further output of NIntegrate::slwcon will be suppressed during this calculation. More…
```

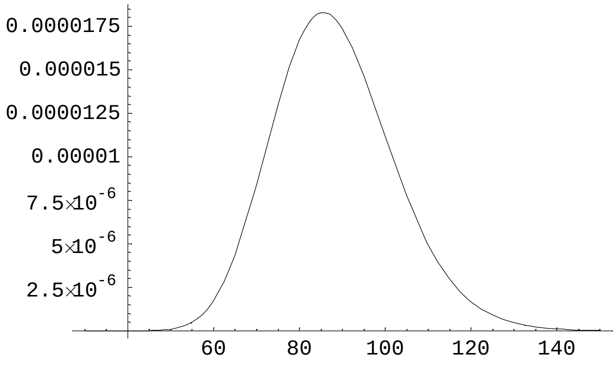


```
Out[46]= - Graphics -
```

That is inconveniently slow, so once again we approximate with an interpolating function. This function automates the process, returning an interpolating function on *n* only, normalized to integrate to one.

```
In[47]:= normInterp[logInterp3D_] := Module[{grid, interp, norm},
            grid = Table[{n, integrateOverError[n, logInterp3D]},
              {n, nMin, nMax, (nMax - nMin) / 100}];
            interp = Interpolation[grid];
            norm = NIntegrate[interp[n], {n, nMin, nMax}];
            grid = grid . {{1, 0}, {0, 1 / norm}};
            Return[Interpolation[grid]]
          ];
```

```
In[48]:= interp1Dme = normInterp[logInterp3Dme];
         interp1Dmf = normInterp[logInterp3Dmf];
```

```
NIntegrate::slwcon :
 Numerical integration converging too slowly; suspect one of the following: singularity, value
   of the integration being 0, oscillatory integrand, or insufficient WorkingPrecision. If your
   integrand is oscillatory try using the option Method->Oscillatory in NIntegrate. More…

NIntegrate::slwcon :
 Numerical integration converging too slowly; suspect one of the following: singularity, value
   of the integration being 0, oscillatory integrand, or insufficient WorkingPrecision. If your
   integrand is oscillatory try using the option Method->Oscillatory in NIntegrate. More…

NIntegrate::slwcon :
 Numerical integration converging too slowly; suspect one of the following: singularity, value
   of the integration being 0, oscillatory integrand, or insufficient WorkingPrecision. If your
   integrand is oscillatory try using the option Method->Oscillatory in NIntegrate. More…

General::stop : Further output of NIntegrate::slwcon will be suppressed during this calculation. More…

NIntegrate::ncvb : NIntegrate failed to converge to prescribed accuracy
   after 13 recursive bisections in errM near {errM, errC} = {0.0155011, 0.018125}. More…

NIntegrate::slwcon :
 Numerical integration converging too slowly; suspect one of the following: singularity, value
   of the integration being 0, oscillatory integrand, or insufficient WorkingPrecision. If your
   integrand is oscillatory try using the option Method->Oscillatory in NIntegrate. More…

NIntegrate::slwcon :
 Numerical integration converging too slowly; suspect one of the following: singularity, value
   of the integration being 0, oscillatory integrand, or insufficient WorkingPrecision. If your
   integrand is oscillatory try using the option Method->Oscillatory in NIntegrate. More…

NIntegrate::slwcon :
 Numerical integration converging too slowly; suspect one of the following: singularity, value
   of the integration being 0, oscillatory integrand, or insufficient WorkingPrecision. If your
   integrand is oscillatory try using the option Method->Oscillatory in NIntegrate. More…

General::stop : Further output of NIntegrate::slwcon will be suppressed during this calculation. More…
```

Here are the two posterior distributions, plus statistics on the distributions.

```
In[50]:= Plot[{interp1Dme[n], interp1Dmf[n]}, {n, nMin, nMax}, PlotRange → All];
         stats[interp1Dme]
         stats[interp1Dmf]
```

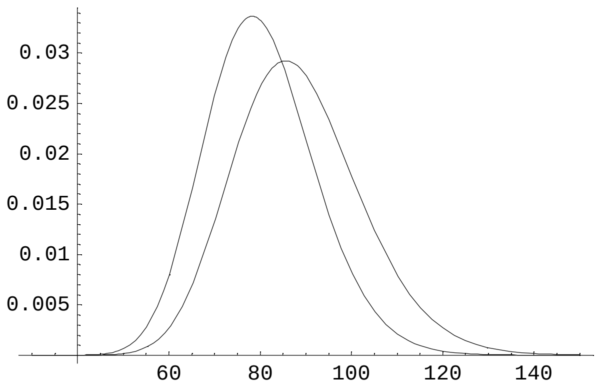


```
Out[51]= {{mean → 88.3617}, {median → 87.4352},
          {mode → 85.6101}, {lower → 63.6765}, {upper → 118.367}}

Out[52]= {{mean → 80.2849}, {median → 79.5943},
          {mode → 78.2272}, {lower → 58.6801}, {upper → 105.824}}
```

The peak on the left is for the fry. The fry appear to have a lower *n* value than the eggs, as would be expected if there were an additional genome number bottleneck between the egg and fry stages, but the degree of overlap between the distributions shows us that the difference is not significant. We can also demonstrate this explicitly:

```
In[53]:= differencePDF[diff_, fx_InterpolatingFunction, fy_InterpolatingFunction] :=
           With[{xMin = fx[[1, 1, 1]], xMax = fx[[1, 1, 2]], yMin = fy[[1, 1, 1]],
             yMax = fy[[1, 1, 2]]}, NIntegrate[fx[x] * fy[x - diff],
             {x, Max[xMin, yMin + diff], Min[xMax, yMax + diff]}]
           ];

In[54]:= Plot[differencePDF[diff, interp1Dme, interp1Dmf],
           {diff, -50, 70}, PlotRange → All];
```

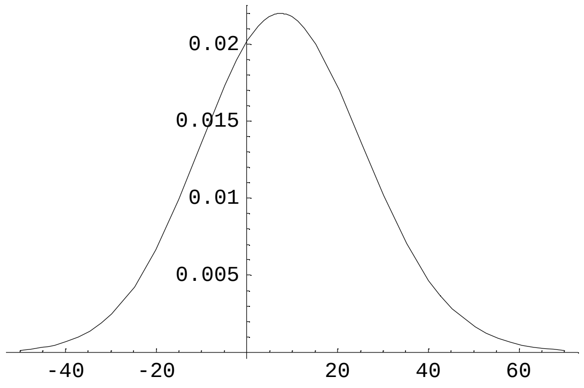

And some stats on that distribution:

```
In[55]:= diffInterpN = Interpolation[Table[
             {diff, differencePDF[diff, interp1Dme, interp1Dmf]}, {diff, -50, 70, 1}]];
         stats[diffInterpN]

Out[56]= {{mean → 8.06378}, {median → 7.88087},
          {mode → 7.39253}, {lower → -27.3068}, {upper → 45.3388}}
```

The 95% confidence interval includes a difference of zero, so we cannot conclude that the two N values differ. The one-sided p-value is:

```
In[57]:= NIntegrate[diffInterpN[n], {n, -50, 0}]

Out[57]= 0.331326
```

We can also apply this methodology to the σ distributions. In particular, are the measurement errors for fry and eggs significantly different?

```
In[58]:= diffInterpσ =
           Interpolation[Table[{diff, differencePDF[diff, σPDFeData, σPDFfData]},
             {diff, -0.005, 0.015, 0.0002}]];
         Plot[diffInterpσ[d], {d, -0.005, 0.015}];
         stats[diffInterpσ]
```

```
NIntegrate::slwcon :
 Numerical integration converging too slowly; suspect one of the following: singularity, value
   of the integration being 0, oscillatory integrand, or insufficient WorkingPrecision. If your
   integrand is oscillatory try using the option Method->Oscillatory in NIntegrate. More…

NIntegrate::ncvb : NIntegrate failed to converge to prescribed
   accuracy after 7 recursive bisections in x near x = 0.011542968750000002`. More…

NIntegrate::slwcon :
 Numerical integration converging too slowly; suspect one of the following: singularity, value
   of the integration being 0, oscillatory integrand, or insufficient WorkingPrecision. If your
   integrand is oscillatory try using the option Method->Oscillatory in NIntegrate. More…

NIntegrate::ncvb : NIntegrate failed to converge to prescribed
   accuracy after 7 recursive bisections in x near x = 0.0115953125`. More…

NIntegrate::ncvb : NIntegrate failed to converge to prescribed
   accuracy after 7 recursive bisections in x near x = 0.01422734375`. More…

General::stop : Further output of NIntegrate::ncvb will be suppressed during this calculation. More…

NIntegrate::slwcon :
 Numerical integration converging too slowly; suspect one of the following: singularity, value
   of the integration being 0, oscillatory integrand, or insufficient WorkingPrecision. If your
   integrand is oscillatory try using the option Method->Oscillatory in NIntegrate. More…

General::stop : Further output of NIntegrate::slwcon will be suppressed during this calculation. More…
```

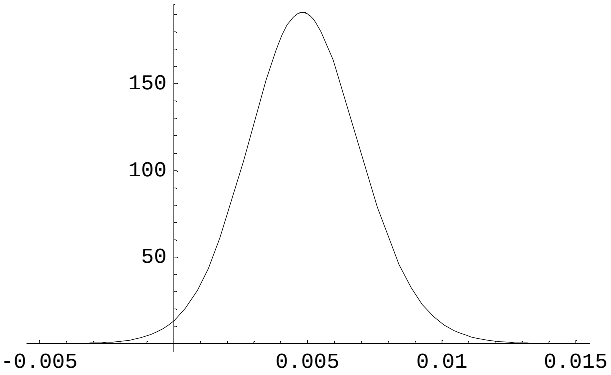


```
Out[60]= {{mean → 0.00487955}, {median → 0.00485638},
           {mode → 0.00480236}, {lower → 0.000700765}, {upper → 0.00922606}}
```

Zero is not in the confidence interval, so we can reject the hypothesis that the measurement errors are the same. The one-sided p-value is

```
In[61]:= NIntegrate[diffInterpσ[d], {d, -0.005, 0}]

Out[61]= 0.0117152
```

xxxxxxxxxxxxxxxxxxxxxxxxxxxxxxxxxxxxxxxxxxxxxxxxxxxxxxxxxxxxxxxxxxxxxxxxxxxxxx
This saves the state of *Mathematica*, so you can avoid waiting 20 minutes while it recalculates. You can restore the state with the command
<< "fish_mitochondria_analysis.mx"

```
In[62]:= DumpSave["fish_mitchondria_analysis.mx"];
```
