# Supplementary material for: The Strength and Timing of the Mitochondrial Bottleneck in Salmon Suggests a Conserved Mechanism in Vertebrates
Source: PLoS One. 2011 May 31;6(5):e20522. doi: 10.1371/journal.pone.0020522 (PMC3105079; doi:10.1371/journal.pone.0020522)
Supplement: Table S1 — Primer and target sequences and dispensation order for pyrosequencing to determine allele frequencies for two heteroplasmic sites. (DOC) [file pone.0020522.s004.doc]

Table S1: Primer and target sequences and dispensation order for pyrosequencing to determine allele frequencies for two heteroplasmic sites.

| Heteroplasmy | 4149^1^ | 4316^1^ |
| --- | --- | --- |
| Forward PCR primer^2^ | CTTCCCCCTTTCTATTCCTCG | CCGAGCAGTAGCACAAACCATTT |
| Reverse PCR primer | GCTCGGAGGGCTCCAATTA | GGCCACGTTGAAGGTTTGAA |
| Sequencing primer | TGCAAGTACAAATAGTACCC | GAGTAAACCCCCCCG |
| Sequence to analyze | CC/TAGGTTAAGATCTGTAAC | TGA/GTGATAATCACGCTAAGTA |
| Dispensation order | GCTCAGTAG | CTGACTGAT |
| ^1^nucleotide position; ^2^ biotinylated | | |
