# Supplementary material for: The Strength and Timing of the Mitochondrial Bottleneck in Salmon Suggests a Conserved Mechanism in Vertebrates
Source: PLoS One. 2011 May 31;6(5):e20522. doi: 10.1371/journal.pone.0020522 (PMC3105079; doi:10.1371/journal.pone.0020522)
Supplement: Table S2 — Raw data for a.mothers (fin clips, additional repeat measurements (1–10) to infer measurement error in founder females), b.oocytes and c.offspring. Frequencies are expressed as x(101). More than one number in one cell indicates repeat measurements for that sample. (DOC) [file pone.0020522.s005.doc]

Table S2. Raw data for a.mothers (fin clips, additional repeat measurements (1-10) to infer measurement error in founder females), b.oocytes and c.offspring. Frequencies are expressed as x(10^1^). More than one number in one cell indicates repeat measurements for that sample.

a. Mothers

| **Family** | **214** | **256** | **263** | **272** | **357** |
| --- | --- | --- | --- | --- | --- |
| **Allele** | **4149 C** | **4149 C** | **4149 C** | **4316 G** | **4316 G** |
| **Original** | 634 654 637 | 330 318 326 | 672 670 678 | 246 237 259 | 303 316 292 |
| **1** | 627 | 333 | 668 | 200 | 254 |
| **2** | 632 | 339 | 672 | 199 | 298 |
| **3** | 616 | 302 | 695 | 194 | 268 |
| **4** | 630 | 338 | 679 | 196 | 283 |
| **5** | 638 | 315 | 679 | 210 | 294 |
| **6** | 645 | 316 | 669 | 179 | 243 |
| **7** | 639 | 333 | 683 | 156 | 256 |
| **8** | 616 | 308 | 676 | 192 |  |
| **9** | 636 | 341 | 676 | 169 |  |
| **10** | 630 | 310 | 681 |  |  |
| **Mean** | 633 | 324 | 677 | 203 | 281 |

b.Oocytes

| **Family** | **214** | **256** | **263** | **272** | **357** |
| --- | --- | --- | --- | --- | --- |
| **Allele** | **4149 C** | **4149 C** | **4149 C** | **4316 G** | **4316 G** |
| **e1** |  |  | 591 | 323 | 300 |
| **e2** |  | 374 | 696 | 283 253 255 | 263 |
| **e3** | 597 |  |  | 222 | 343 |
| **e4** | 630 |  |  | 228 |  |
| **e5** | 654 |  | 630 | 236 | 340 314 345 |
| **e6** | 597 |  |  | 199 |  |
| **e7** | 578 611 |  |  | 273 258 240 | 284 |
| **e8** | 690 | 357 370 323 |  | 155 | 325 259 239 |
| **e9** | 500 | 329 |  | 182 |  |
| **e10** | 608 597 589 |  |  | 197 147 171 | 236 |
| **e11** | 574 571 578 | 332 | 699 | 239 | 254 |
| **e12** | 669 637 654 | 394 | 675 | 150 | 277 |
| **e13** | 667 653 636 | 337 | 664 | 151 | 343 |
| **e14** | 770 758 772 | 409 | 716 | 203 | 153 |
| **e15** | 638 628 605 | 434 | 659 | 228 | 240 |
| **e16** | 680 648 649 | 401 | 643 | 175 | 211 |
| **e17** | 627 641 625 | 395 | 675 | 208 | 216 |
| **e18** | 566 564 563 | 341 | 736 | 236 | 224 |
| **e19** | 638 624 616 | 358 | 726 | 183 | 310 |
| **e20** | 666 664 674 | 461 | 676 | 169 | 214 |
| **e21** | 651 629 615 | 274 |  |  |  |
| **e22** | 686 660 685 | 340 |  |  |  |
| **e23** | 686 670 656 | 363 |  |  |  |
| **e24** | 710 679 700 | 408 |  |  |  |
| **e25** | 656 656 640 | 397 |  |  |  |
| **e26** | 725 704 708 | 340 |  |  |  |
| **e27** | 621 633 631 | 395 |  |  |  |
| **e28** | 672 653 649 | 339 |  |  |  |
| **e29** | 647 649 633 | 383 |  |  |  |
| **e30** | 634 612 621 | 441 |  |  |  |
| **Mean** | 638 | 374 | 676 | 209 | 263 |

c. Offspring

| **Family** | **214** | **256** | **263** | **272** | **357** |
| --- | --- | --- | --- | --- | --- |
| **Allele** | **4149 C** | **4149 C** | **4149 C** | **4316 G** | **4316 G** |
| **f1** | 649 | 397 399 404 | 765 | 276 | 248 279 278 |
| **f2** | 736 | 287 | 698 697 700 | 273 | 408 |
| **f3** | 736 744 752 | 332 | 648 | 226 220 234 | 278 |
| **f4** | 749 | 313 297 344 | 695 | 190 | 288 269 278 |
| **f5** | 640 | 334 | 669 | 152 | 310 |
| **f6** | 628 635 641 | 333 | 729 | 181 188 189 | 334 |
| **f7** | 618 | 338 | 652 637 658 | 243 | 275 |
| **f8** | 597 | 311 | 694 | 173 | 369 |
| **f9** | 640 | 329 344 327 | 642 | 254 | 330 296 303 |
| **f10** | 606 | 413 | 708 681 702 | 154 | 267 |
| **f11** | 654 668 642 | 430 | 731 | 166 | 339 |
| **f12** | 600 585 576 | 353 | 720 | 144 | 294 |
| **f13** | 609 610 588 | 445 | 660 | 149 | 288 |
| **f14** | 690 684 690 | 340 | 724 | 180 | 270 |
| **f15** | 637 641 639 | 355 | 712 | 242 | 294 |
| **f16** | 717 | 362 | 763 | 152 | 347 |
| **f17** | 485 | 428 | 658 | 201 | 359 |
| **f18** | 692 | 368 | 722 | 212 | 357 |
| **f19** | 703 | 351 | 685 | 139 | 278 |
| **f20** | 622 | 403 | 714 | 240 | 389 |
| **Mean** | 650 | 362 | 699 | 198 | 316 |
